# FGF23 Expression Is a Promising Immunohistochemical Diagnostic Marker for Undifferentiated Pleomorphic Sarcoma of Bone (UPSb)

**DOI:** 10.3390/genes15020242

**Published:** 2024-02-14

**Authors:** Hafid O. Al-Hassi, Naser M. Ali, Hannah Cooke, Shamini De Silva, Anna T. Brini, Pavithra Babu, Vaiyapuri Sumathi, Mark R. Morris, Stefania Niada

**Affiliations:** 1 Research Institute of Healthcare Science, Faculty of Science and Engineering, University of Wolverhampton, Wolverhampton WV1 1LY, UK; h.cooke2@wlv.ac.uk (H.C.); m.r.morris2@wlv.ac.uk (M.R.M.); 2 Institute of Cancer and Genomic Sciences, College of Medical and Dental Sciences, University of Birmingham, Edgbaston, Birmingham B15 2TT, UK; nasermali@hotmail.com; 3 Department of Medical Laboratories, General Ahmadi Hospital (KOC Hospital), Ahmadi 61008, Kuwait; 4 Laboratory of Biotechnological Applications, IRCCS Orthopedic Institute Galeazzi, 20157 Milan, Italy; anna.brini@unimi.it; 5 Department of Biomedical, Surgical and Dental Sciences, University of Milan, 20129 Milan, Italy; 6 Acute Medicine, Birmingham Heartlands Hospital, University Hospital Birmingham, Birmingham B9 5SS, UK; pavithra.babu@uhb.nhs.uk; 7 Department of Musculoskeletal Pathology, University Hospital of Birmingham, Birmingham B15 2TT, UK; vaiyapuri.sumathi@nhs.net

**Keywords:** undifferentiated high-grade pleomorphic sarcoma of bone, osteosarcoma, dedifferentiated chondrosarcoma, FGF23, immunohistochemistry, molecular marker

## Abstract

**Background:** Undifferentiated pleomorphic sarcoma of bone (UPSb) is a rare primary bone sarcoma that lacks a specific line of differentiation. Distinguishing between UPSb and other malignant bone sarcomas, including dedifferentiated chondrosarcoma and osteosarcoma, is challenging due to their overlapping features. We have previously identified that UPSb tumours have elevated mRNA levels of Fibroblast Growth Factor 23 (*FGF23*) transcripts compared to other sarcomas including osteosarcoma. In the present study, we evaluated the specificity and practicality of FGF23 immunoreactivity as a specific diagnostic tool to differentiate UPSb tumours from osteosarcomas and dedifferentiated chondrosarcomas. **Methods:** A total of 10 UPSb, 10 osteosarcoma, and 10 dedifferentiated chondrosarcoma cases (all high-grade), were retrieved and immunohistochemistry for FGF23 was performed. **Results:** FGF23 protein was expressed at high levels in 80–90% of undifferentiated pleomorphic sarcoma of the bone cases, whereas it was expressed at significantly lower levels in dedifferentiated chondrosarcoma and osteosarcoma cases. A semiquantitative analysis, considering the intensity of immunoreactivity, confirmed significantly elevated FGF23 expression levels in UPSb tissues compared to those observed in osteosarcoma and dedifferentiated chondrosarcoma tissues. **Conclusions:** The results we present here suggest that FGF23 immunohistochemistry may be a useful tool to aid in differentiating UPSb from morphologically similar malignant bone sarcomas, especially in situations where sampling is restricted and there is limited clinical information available.

## 1. Introduction

### 1.1. Undifferentiated Pleomorphic Sarcoma of Bone (UPSb) Features

Undifferentiated high-grade pleomorphic sarcoma of bone (UPSb), previously known as malignant fibrous histiocytoma of bone (MFH), is a rare malignant neoplasm that represents less than 2% of all primary malignant bone tumours. Patients typically present with painful, regional masses. It typically occurs between the ages of 20 and 80, with the highest incidence observed in the second to fourth decades of life. UPSb encompasses pleomorphic sarcomas that cannot be classified into any other bone tumour category, lacking a specific line or pattern of differentiation. These tumours are highly aggressive, with an overall 5-year survival rate reported to range between 30% and 60% [1]. Undifferentiated pleomorphic sarcoma of bone commonly arises in the long bones of the lower extremities (primarily distal femur, proximal tibia) and has a high tendency to metastasise, particularly to the lungs (~50%) [2]. Risk factors for UPSb are bone infarct, Paget disease, or prior radiation. UPSb lesions are heterogeneous and, morphologically, composed of atypical spindled and pleomorphic cells that can be arranged in a storiform, fascicular, or haphazard pattern [3]. Current strategies for UPSb treatment include neoadjuvant chemotherapy, followed by surgical resection and adjuvant chemotherapy. UPSb may share clinical presentations and morphological similarities with two other malignant bone lesions, osteosarcoma and dedifferentiated chondrosarcoma.

### 1.2. Osteosarcoma (OS) Features

Osteosarcoma is the most common primary malignancy of the bone, characterised by the deposition of an immature osteoid matrix [4]. There are two incidence peaks: the first occurs typically between 10 and 14 years old and the second manifests in adults aged 65 years and older. It typically arises in the metaphysis of an extremity’s long bone, usually around the knee [5,6]. In adults, it can also originate in the axial skeleton, pelvis, or craniofacial bones. Risk factors for osteosarcoma include previous radiation therapy, Paget disease of bone, and germline abnormalities [7]. The overall 5-year survival rate for osteosarcoma is 70%. Metastatic disease is the major cause of osteosarcoma-related death. There are different types of treatment for patients with osteosarcoma. In addition to neoadjuvant chemotherapy, followed by surgical resection and adjuvant chemotherapy, radiation therapy can be used in combination with other treatments. Moreover, samarium or targeted therapy with kinase or mammalian target of rapamycin inhibitors can be used in recurrent and metastatic OS.

### 1.3. Dedifferentiated Chondrosarcoma (DDSC) Features

Chondrosarcoma is the second most common sarcoma of bone worldwide, following osteosarcoma. It typically occurs between the ages of 30 and 60 [8]. Dedifferentiated chondrosarcoma is a rare subtype of chondrosarcoma that is a primary cartilaginous malignant neoplasm. It accounts for only 1–2% of all bone malignancies and 10 to 20% of all chondrosarcomas. This form of cancer can develop from an existing chondrosarcoma. Notably, there is an estimated risk of dedifferentiation in 7–20% of cases involving conventional chondrosarcoma. Dedifferentiated chondrosarcoma predominantly occurs in the appendicular skeleton, and it has a higher likelihood of manifestation in the lower limb. Common sites include the femur, pelvis, and humerus, as well as the scapula, rib, and tibia. Additionally, it may affect other bone types, with a predilection for pelvic bones and scapulae [9]. It is usually characterised by two distinctive histopathological components: a low-grade tumour, characterised by the deposition of nonosseous hyaline cartilage matrix, closely intersected with a high-grade noncartilaginous sarcoma tumour. The high-grade component of dedifferentiated chondrosarcoma can exhibit characteristics associated with various sarcomas, including osteosarcoma and UPSb. Misdiagnosis may occur when dedifferentiated chondrosarcoma is mistaken for a sarcoma representing the undifferentiated component, especially if only the high-grade component is evident in the smears. There are currently no established guidelines for treating advanced-stage patients with dedifferentiated chondrosarcoma. Better outcomes are associated with wide surgical margins, as they lead to higher survival rates and a decreased risk of recurrence. Palliative treatment options, such as immunotherapy and chemotherapy, are employed, even though dedifferentiated chondrosarcoma tends to exhibit relative resistance to these therapies [8,9]. Dedifferentiated chondrosarcoma is associated with a poor prognosis and an aggressive course, leading to a 5-year survival rate of 7% to a maximum of 24%. The median survival range is 7 to 15 months [10].

### 1.4. Diagnostic Procedures, Markers, and Challenges

The diagnosis of UPSb, osteosarcoma, and dedifferentiated chondrosarcoma necessitates a comprehensive medical history, coupled with multiple imaging tests of the tumour and potential metastatic sites (e.g., X-ray, magnetic resonance imaging, computed tomography scan, and positron emission tomography), as well as a thorough histological examination. From a histopathological perspective, distinguishing between osteosarcoma and UPSb relies on identifying the presence of focal osteoid, which is characteristic of osteosarcoma and absent in UPSb [2]. Conversely, differentiating between UPSb and dedifferentiated chondrosarcoma relies on the presence of a low-grade cartilaginous component in the latter cancer type [11]. In biopsy or limited sampling, differentiating between UPSb, osteosarcoma, and dedifferentiated chondrosarcoma can be challenging. Isocitrate dehydrogenase (*IDH*) mutations are a useful marker that supports the diagnosis of dedifferentiated chondrosarcoma rather than UPSb. Indeed, *IDH1/2* mutations are frequent in dedifferentiated chondrosarcoma, while they are absent in UPSb and osteosarcoma [3]. In addition to this, other molecular tests to distinguish between the three bone lesions are still required for diagnosis. UPSb, OS, and DDCS not only exhibit common features, especially in the high-grade component, but also share certain immunohistochemical markers and genetic mutations. For instance, SOX-9 is positively expressed in DDCS, but it can also be observed in OS and various other types of sarcomas, posing challenges in differential diagnosis. Furthermore, *TP53* is among the most frequently mutated genes in all three types of sarcomas. Recently, we explored the genomic and transcriptomic landscape of UPSb and identified mutations in *TP53* and chromatin-remodelling-related genes (*H3F3A*, *ATRX*, and *DOT1L*) in 29% and 36% of UPSb samples, respectively [12].

### 1.5. FGF23 Expression in UPSb

In our earlier investigation on the genomic and transcriptomic characterisation of undifferentiated pleomorphic sarcoma of bone, RNA sequencing data indicated elevated transcriptomic expression of *FGF23* (fibroblast growth factor 23) in the UPSb in comparison with other sarcomas, including osteosarcoma. This elevated expression of *FGF23* was confirmed in four UPSb tumours using RT-qPCR and, potentially, can be of diagnostic importance [12]. The objectives of this study were to determine whether the FGF23 protein is highly expressed in UPSb and to investigate the potential of immunohistochemistry as a practical and cost-effective method for identifying differential expression of FGF23. Following this, in the present study, we evaluated the specificity and practicality of FGF23 immunoreactivity as a specific diagnostic tool to differentiate UPSb tumours from osteosarcomas and dedifferentiated chondrosarcomas.

## 2. Materials and Methods

### 2.1. Study Samples

A total of 30 bone tumour cases, including 10 undifferentiated pleomorphic sarcoma of the bone (UPSb), 10 osteosarcoma, and 10 dedifferentiated chondrosarcoma cases (all high-grade), were identified through a retrospective search of the pathology database at the Royal Orthopaedic Hospital NHS Foundation Trust Tumour Bank. SV (pathologist) confirmed the pathology using well-established criteria. All samples were obtained with patients’ informed consent and ethical approval (NRES—Staffordshire Research ethics committee, Ref:12/EM/0048, approval date: 15 December 2014). All samples were pseudonymised and used in alliance with the ethical rules and regulations presented in the Declaration of Helsinki. Sample details are summarised below and reported in Table 1.

For UPSb, the age of the patients ranged between 20 and 86 years old at the time of diagnosis. Of these patients, seven were women, and three were men. The tumour size ranged between 6 and 13 cm. The tumours occurred at the femur, tibia, humerus, and fibula. The tumours’ morphology included spindle (n = 10), pleomorphic (n = 1), spindle and epithelioid (n = 2), and spindle and pleomorphic cells (n = 1).

For osteosarcomas, the age of the patients ranged between 9 and 69 years old. Of these patients, five were men, and five were women. The tumour size ranged between 7 and 16 cm. The sarcomas occurred at the femur, tibia, and humerus.

For dedifferentiated chondrosarcoma, the age of the patients ranged between 51 and 78 years old. Of these patients, seven were men, and three were women. The tumour size ranged between 7 and 31 cm. The anatomical sites included the femur, scapula, pelvis, and sternum.

### 2.2. FGF23 Immunohistochemistry

Paraffin-embedded tumour tissue sections were dewaxed in xylol and rehydrated in descending series of ethanol to water. Endogenous enzymes were blocked with 15% H_2_O_2_ in methanol for 15 min, then washed in tap water. Antigens were retrieved via heat induction and nonspecific binding was blocked using normal rabbit serum for 1 h in a humidified chamber. Anti-FGF23 (AB192497, Abcam, Cambridge, UK) antibody was added at 1:50 dilution overnight at 4 °C. Slides were washed, and biotinylated secondary antibody was added (30 min). Avidin–biotin complex was used, and staining was visualised using 3,3′-diaminobenzidine. Counterstaining was performed using haematoxylin. Omitting the primary antibody was used as a control. Images were taken using an Olympus BX51 microscope (Olympus, Southend-on-Sea, UK). Analysis was performed by two assessors blind to the treatment under 20× and 40× magnification. Semiquantitative analysis was performed based upon the intensity of immunoreactivity and scored from 0 to 3 (0 = 1–25%,1 = 25–50%, 2 = 50–75%, 3 = 75–100%). Five high-magnification fields were assessed per sample. An average immunoreactivity score was calculated. An independent assessor resolved discrepancies between scores.

### 2.3. Statistical Analysis

The statistical significance of differences between experimental groups was determined using two-tailed unpaired *t* tests, and *p* < 0.05 was considered significant.

## 3. Results

**Figure 1:**
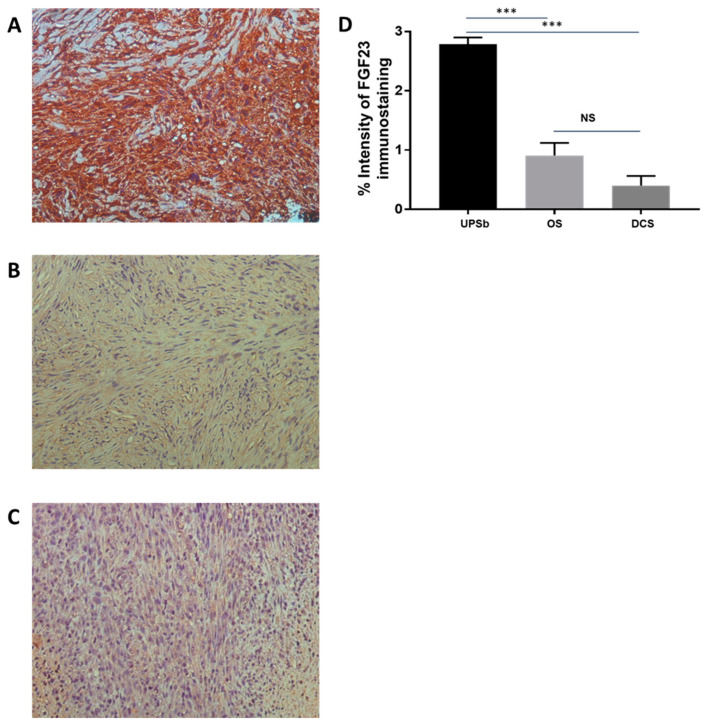
FGF23 (fibroblast growth factor 23) immunoreactivity (brown colour) and expression. (**A**) Intense staining in spindle-shaped cells of UPSb tissue. (**B**) Weak staining in spindle-shaped cells of osteosarcoma tissue. (**C**) Weak staining in spindle-shaped cells of dedifferentiated chondrosarcoma tissue ((**A**–**C**) magnification: 20×). (**D**) FGF23 expression is significantly higher in UPSb tumour tissues (n = 10) compared with that in osteosarcoma (OS) (n = 10) and dedifferentiated chondrosarcoma (DCS) (n = 10) tissues. Paired *t* test was used, and *p* values of <0.05 were considered statistically significant (*** *p* < 0.0001).

Approximately 80–90% of undifferentiated pleomorphic sarcoma of the bone (UPSb) tissue sections expressed FGF23 with high intensity (Figure 1A). In contrast, FGF23 staining was weak and patchy in tissue sections from both osteosarcoma (Figure 1B) and dedifferentiated chondrosarcoma (Figure 1C). Semiquantitative analysis based on the intensity of the immunoreactivity confirmed that FGF23 expression levels were significantly higher in UPSb tissues than in osteosarcoma and dedifferentiated chondrosarcoma tissues (Figure 1D). There was no correlation between the immunostaining and the previously identified mutations in *TP53*, *H3F3A*, *ATRX*, and *DOT1L* [12].

**Figure 2:**
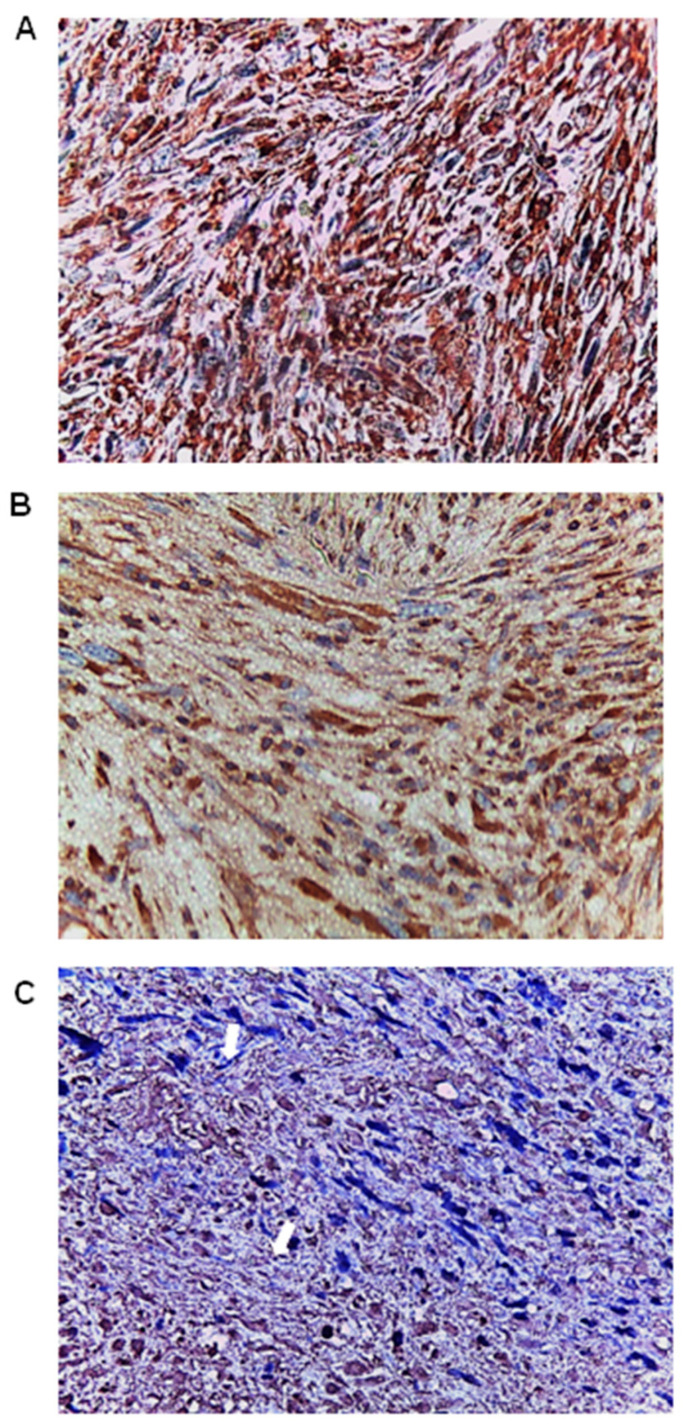
FGF23 (fibroblast growth factor 23) expression in UPSb tumour tissues (n = 10). (**A**) The immunostaining was mainly cytoplasmic throughout the UPSb tissues. (**B**) Intensity of the staining was higher in the spindle-shaped cells located in the cellular-dense lesions of UPSb. (**C**) Nuclear immunostaining in some UPSb pleomorphic cells (arrows) ((**A**–**C**) magnification: 40×).

In UPSb tissues, the immunostaining was predominantly cytoplasmic, granular, and uniformly distributed throughout the tissues. The intensity of the staining was higher in the spindle-shaped cells located in the cellular-dense lesions (Figure 2A). However, strong nuclear immunostaining was also detectable in some pleomorphic cells (Figure 2B,C).

**Figure 3:**
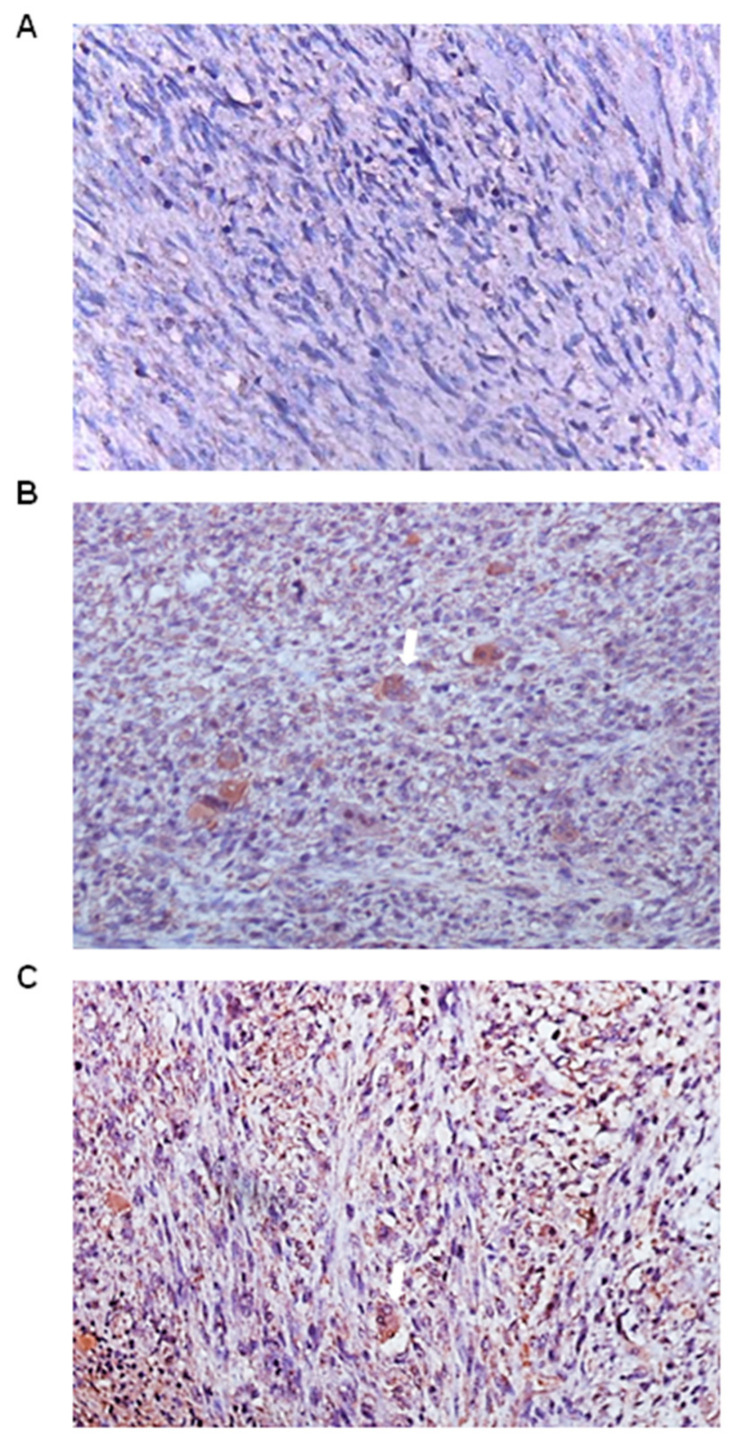
FGF23 (fibroblast growth factor 23) expression in osteosarcoma tumour tissues (n = 10). (**A**) Moderate-to-weak immunostaining in all types of cells in the osteosarcoma tissue sections. (**B**) Moderate immunostaining localised to large, atypical cells (arrow). (**C**) Moderate immunostaining was observed in the osteoclast-like giant cells (arrow) (magnification: 20×).

The expression of FGF23 levels was low in osteosarcoma in terms of both cell positivity and the intensity of the immunostaining (Figure 3A). However, large, atypical cells were scattered throughout the tissue and stained moderately for FGF23 (Figure 3B). Immunohistochemical staining was also observed in the osteoclast-like giant cells (Figure 3C).

**Figure 4:**
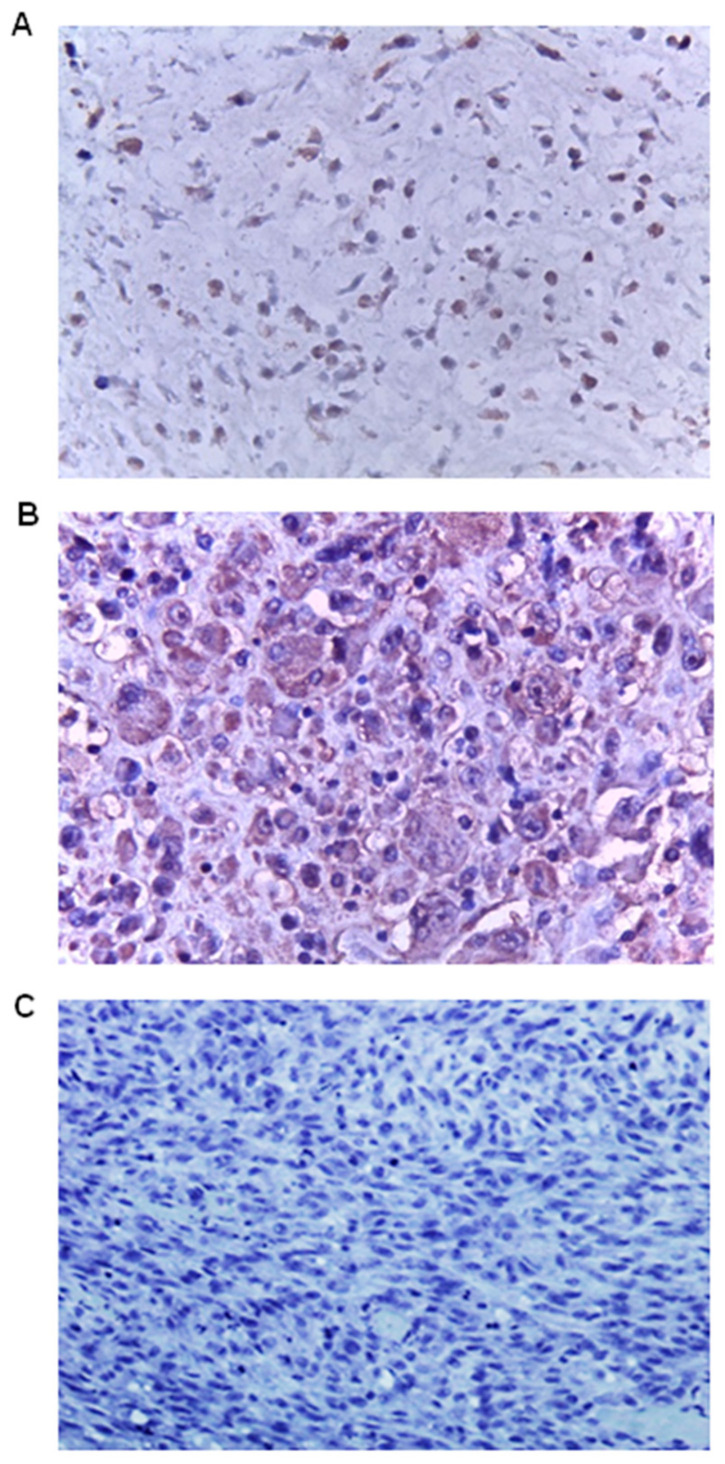
FGF23 (fibroblast growth factor 23) expression in dedifferentiated chondrosarcoma tumour tissues (n = 10). (**A**) Mild-to-weak staining mainly in the cytoplasm. (**B**) Weak staining was localised to giant cells ((**A**,**B**) magnification: 40×). (**C**) Absence of FGF23 primary antibody was used as a negative control and indicated the specificity of the immunostaining (magnification: 20×).

Although some dedifferentiated chondrosarcoma tissue sections were stained for FGF23, this staining was mild to weak and mainly in the cytoplasm (Figure 4A). Giant cells scattered throughout the tissues and were also stained weakly for FGF23 (Figure 4B).

## 4. Discussion

Undifferentiated pleomorphic sarcoma of the bone, dedifferentiated chondrosarcoma, and osteosarcoma share many similarities in presentation and morphology, making accurate diagnosis challenging [5]. Correct diagnosis is extremely important as dedifferentiated chondrosarcomas are significantly more aggressive and respond poorly to chemotherapeutic treatment [13]. Conversely, chemotherapy administration has been identified as a favourable prognostic factor, and it significantly improves patient 5-year disease-specific survival in UPSb [11,14]. Molecular tests are useful tools for facilitating accurate diagnosis, especially when clinical information is limited or radiologic findings are inconclusive. *IDH* mutations were identified as frequent in dedifferentiated chondrosarcoma but absent in the other two bone lesions. Furthermore, in a previous study conducted by our team, *FGF23* was identified as a gene specifically expressed in UPSb tumours [12]. This finding surfaced during a supervised expression analysis that compared UPSb to other sarcoma subtypes, including osteosarcoma. Here, we showed that, when comparing these three tumour types, FGF23 protein is expressed at high levels specifically in UPSb. This indicates a relevant concordance of gene and protein expression in UPSb that is not obvious. The synthesis and maintenance of cellular proteins entail a complex and interconnected series of processes. This encompasses the transcription, processing, and degradation of messenger RNAs (mRNAs), as well as the translation, localisation, modification, and programmed degradation of the proteins they encode. The levels of proteins within the cell are indicative of a dynamic equilibrium maintained through the intricate interplay of these processes. In a recent study, gene and protein expression in cancer tissues was analysed, and it was revealed that gene and protein expression was uncorrelated for some biomarkers [15]. Conversely, in our scenario, the elevated expressions of both the *FGF23* gene and its corresponding protein suggest that measurements of both gene and protein expressions could potentially serve as biomarkers for UPSb. Moreover, through the integration of this study with our previous research, the distinction in FGF23 expression between UPSb and OS becomes evident with two distinct cohorts of osteosarcoma. Specifically, here, we detected a significant difference between UPSb and 10 osteosarcoma cases from the Royal Orthopaedic Hospital NHS Foundation Trust Tumour Bank in terms of FGF23 protein expression. In our previous study, increased *FGF23* gene expression was coupled with the distinction between 14 UPSb and 16 osteosarcoma samples randomly chosen from the SRA bioproject PRJNA345424, which was revealed via unsupervised hierarchical clustering. The low level of FGF23 expression in dedifferentiated chondrosarcoma and osteosarcoma is consistent with the findings of a previous report [16]. FGF23 is a bone-derived hormone mainly secreted by osteocytes and osteoblasts. FGF23 binds to target cells that express the FGF receptor-1 and, in most cases, the co-receptor α klotho. α klotho is highly expressed in the distal convoluted tubules of the kidney where FGF23-α klotho-FGFR signalling regulates phosphate and calcium homeostasis and suppresses the synthesis of 1,25(OH)_2_D_3_, active vitamin D [17]. In addition to bone cells, FGF23 can also be produced by tumour cells. As such, tumour-associated overexpression of FGF23 can result in tumour-induced osteomalacia [18] or oncogenic hypophosphatemic osteomalacia. In this paraneoplastic syndrome, the increased production of FGF23, in line with its main endocrine effects, induces renal phosphate excretion and reduction of 1,25(OH)_2_D_3_, causing osteomalacia and demineralised bone [19]. Phosphaturic mesenchymal tumours are most frequently responsible for tumour-induced osteomalacia [20]. FGF23 can be also produced by malignancies including colon adenocarcinoma, ovarian cancer, small cell carcinoma of the lung, anaplastic thyroid carcinoma, B-cell non-Hodgkin lymphoma, breast cancer, and intracranial tumours. In patients with bone metastasis due to different solid tumours, a higher FGF23 plasma concentration is associated with shorter survival and shorter time to skeletal-related events. Curiously, no tumour-induced osteomalacia has been reported in association with UPSb. Similar observations were noted in ovarian cancer patients, in whom both serum levels and immunohistochemistry staining of FGF23 correlated with an advanced stage of the disease but not with hypophosphatemia [21]. In contrast, for phosphaturic mesenchymal tumours, a direct correlation between FGF23 expression and this paraneoplastic syndrome has been identified [16]. Further examination of both the FGF23 and phosphate serum levels in these patients could provide insights into this aspect. In addition to endocrine effects, FGF23 also exerts paracrine actions. The effects of locally produced FGF23 include the regulation of inflammation in hepatocytes, the induction of cardiac hypertrophy, or the inhibition of neutrophils. Some of these effects are not dependent on klotho [17]. Within the bone microenvironment, FGF23’s role is not completely defined. However, Wang et al.’s in vitro results showed that the overexpression of FGF23 was associated with suppressed osteoblast differentiation and matrix mineralisation [22], independent of systemic effects on phosphate homeostasis. Moreover, other in vitro studies have shown that treating primary calvarial osteoblasts or the osteoblastic MC3T3-E1 cell line with supraphysiological levels of FGF23 results in increased cell proliferation and inhibition of mineralisation, thus confirming FGF23 effects that are serum-phosphate-independent [23,24,25]. This may elucidate some of the phenotypes seen in UPSb, such as the absence of a definitive pattern of differentiation, as well as the lack of chondroid and osteoid matrix deposition and matrix mineralisation [3,5,11]. In contrast, osteosarcoma and dedifferentiated chondrosarcoma, both of which show low FGF23 expression, have high matrix mineralisation [26,27]. Therefore, the phenotypes associated with UPSb may be attributed to the overexpression of FGF23, as these are not seen in osteosarcoma and dedifferentiated chondrosarcoma, which have low FGF23 levels. Nevertheless, it should be mentioned that moderate FGF23 staining was detected in osteosarcoma samples, encompassing both large, atypical cells and osteoclast-like giant cells. Additionally, weak staining for FGF23 was detectable in the giant cells of dedifferentiated chondrosarcoma. FGF23 expression was reported in giant cell tumour tissue; however, the authors did not specify whether the protein was expressed in spindle cells or osteoclast-like giant cells [28]. The presence of giant cells is frequent in the rare osteosarcoma subtype known as telangiectatic osteosarcoma. Further exploration of FGF23 expression in this osteosarcoma subtype and, potentially, other subtypes could enhance the investigation, especially considering the existence of various described osteosarcoma subtypes [29]. Furthermore, single-cell RNA sequencing (scRNA-seq) analysis, potentially employing a targeted approach, could elucidate the expression of FGF23 in distinct subsets of UPSb that have also been previously identified by Yuan et al. [30]. In parallel, the search for FGF23 expression in the complex osteosarcoma microenvironment, which has been recently highlighted by scRNA-seq investigations, could help with understanding if FGF23 is not expressed in any OS and associated normal subpopulation [31,32,33]. Indeed, OS heterogeneity in terms of subtypes, cells of origin [34], and tumour complexity might require further evaluation. Moreover, considering the remodelling that occurs during OS, FGF23 could be checked in OS at different times.

FGF23 also exerts classical protumourigenic effects through autocrine and paracrine mechanisms. In prostate cancer, FGF23 is present at increased levels in tumour tissues. Moreover, it is expressed as an autocrine growth factor in several prostate cancer cell lines. In vitro studies revealed that exogenous FGF23 increases proliferation, invasion, and anchorage-independent growth. Conversely, the suppression of FGF23 in prostate cancer cell lines results in a reduction in these phenotypes [35]. In multiple myeloma (MM), FGF23 is not expressed by cancer cells. Yet, multiple myeloma (MM) cancer cells exhibit a response to FGF23, probably released by bone cells. This response leads to an elevation in heparanase levels, a crucial regulator of metastases and interactions within the tumour microenvironment, the heightened levels of which serve as an indicator of a poor prognosis in MM [36]. Further work is required to determine if FGF23 expression contributes to UPSb tumourigenesis. If this is the case, then FGF23 may be a promising UPSb therapeutic target. The anti-FGF23 monoclonal antibody drug (KRN23) that has been developed to treat tumour-induced osteomalacia [37,38,39] may have the potential to be re-purposed as an anti-UPSb therapeutic. Altogether, our findings suggest that FGF23 could be utilised as a potential specific molecular and/or cellular target in the diagnosis and management of UPSb patients. However, future work should focus on other potential diagnostic markers using scRNAseq.

There are some limitations to this study. As a negative control in immunohistochemistry, an isotype control should be used to confirm the specificity. Moreover, conducting multicentric studies with additional sample cohorts could strengthen our findings. In addition, future work should focus on using qPCR as an accompanying method to confirm the protein expression using IHC. However, in this study, our focus was on using a technique that is more affordable and widely available in pathology laboratories.

## 5. Conclusions

The elevated immunohistochemical expression of FGF23 in undifferentiated pleomorphic sarcoma of the bone suggests that immunohistochemical staining for FGF23 may function as a diagnostic help for this specific type of cancer. Notably, it may contribute to the distinction between UPSb and osteosarcoma or dedifferentiated chondrosarcoma—bone cancers characterised by shared features. This is particularly valuable in situations where sampling is restricted and there is limited clinical information available. These findings warrant a larger study to confirm the current data.

## Figures and Tables

**Table 1:** Clinicopathological features of undifferentiated pleomorphic sarcoma of the bone (UPSb), osteosarcoma (OS), and dedifferentiated chondrosarcoma (DDCS) cohorts. na, not available.

Characteristics	UPSb (n = 10)	OS (n = 10)	DDSC (n = 10)
**Age**			
Mean ± SD	61.0 ± 20.2	36.4 ± 20.3	64.5 ± 20.2
Range	20–86	9–69	51–78
**Sex**			
Female	7 (70%)	5 (50%)	3 (30%)
Male	3 (30%)	5 (50%)	7 (70%)
**Primary site**			
Femur	7 (70%)	5 (50%)	7 (63%)
Tibia	1	4 (40%)	-
Fibula	1	-	-
Humerus	1	1	-
Pelvis/Scapula/Sternum	-	-	1/1/1
**Size (range in mm)**	60–132	75–160	70–310
**Recurrence**			
Yes	0	0	6 (60%)
No	9 (90%)	7 (70%)	4 (40%)
na	1	3	-
**Metastasis**			
Yes	5 (50%)	6 (60%)	6 (64%)
Lung	3 (30%)	3 (30%)	4 (40%)
Chest	1	3 (30%)	1
Other sites	1 (bone, liver)	-	3
No	5 (50%)	3 (30%)	3 (30%)
na	-	1	-
**Chemotherapy**	4 (40%)	9 (90%)	1
**5-Year Survival**			
Yes	8 (80%)	4 (40%)	3 (27%)
No	2 (20%)	5 (50%)	3 (27%)
na	-	1	4

## Data Availability

Data available on request.
